# Central Pontine Myelinolysis with Minimal Hyponatremia in the Setting of AIDS

**DOI:** 10.1155/2015/421923

**Published:** 2015-10-29

**Authors:** Joseph M. Carrington, Galo Sanchez, Jennifer L. Berkeley

**Affiliations:** ^1^Department of Internal Medicine, Johns Hopkins University, Sinai Hospital of Baltimore, Baltimore, MD 21215, USA; ^2^Department of Neurology, Division of Neurocritical Care, Sinai Hospital of Baltimore, Baltimore, MD 21215, USA

## Abstract

Central pontine myelinolysis (CPM) is classically attributed to overly rapid correction of profound hyponatremia. However, there are case reports of this disease in the setting of normal serum sodium or minimal hyponatremia. These cases have been hypothesized to be secondary to other metabolic disturbances such as hyperglycemia or hypophosphatemia. Eunatremic CPM has also been described in patients with advanced acquired immune deficiency syndrome (AIDS). The mortality risk in this special population is significantly higher than those with hyponatremia-associated CPM, but the mechanisms are unclear. We discuss a case of a man with AIDS who developed CPM with minimal hyponatremia and no other metabolic disturbances. Common variables within this population, such as hypoalbuminemia and lymphoma, are discussed as potential factors contributing to the pathophysiology. Reporting these atypical cases is crucial to our understanding of how to prevent future cases.

## 1. Case

A 53-year-old man presented with fevers, night sweats, fatigue, and dry cough. He was diagnosed with acquired immune deficiency syndrome (AIDS) one month earlier, with a CD4^+^ lymphocyte count of 54 cells/mm^3^. He was started on antiviral therapy and infection prophylaxis. Physical examination revealed a thin man who was alert and oriented with bilateral crackles on lung exam and diminished breath sounds on the left. He also had small erythematous macules on his left leg. A chest X-ray showed bilateral pleural effusions and a thoracentesis revealed exudative fluid suggestive of lymphoma on flow cytometry. A biopsy of the leg lesions confirmed intravascular lymphoma and chemotherapy was discussed.

**Figure 1:**
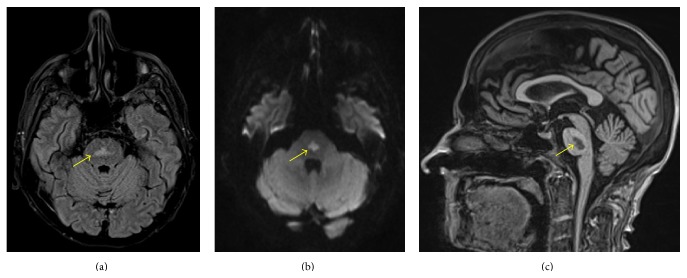
Brain magnetic resonance imaging shows a lesion in the central pons with imaging characteristics consistent with demyelination. The lesion is hyperintense on both (a) Fluid-Attenuated Inversion Recovery Image and (b) Diffusion-Weighted Imaging. The lesion is hypointense on (c) T1-weighted sequence.

On hospital day six, he became acutely dysarthric, ataxic, and delirious and was oriented to name only. Magnetic resonance imaging (MRI) of the head revealed a hyperintense lesion in the central pons on FLAIR/T2-weighted sequences which also exhibited restricted diffusion and was hypointense on T1-weighted images (Figure 1). He was diagnosed with central pontine myelinolysis (CPM). On admission, he had minimal hyponatremia of 129 mmol/L which had corrected slowly over six days to 136 mmol/L. No other electrolyte abnormalities were present. His albumin was low at 1.5 g/dL. On lumbar puncture, CSF fluid appeared clear with one white blood cell/mm^3^, 43 mg/dL of protein, and 44 mg/dL of glucose. Culture and Gram stain were negative, as was an infectious-disease work-up. On hospital day 18, he developed CMV colitis and a colonic perforation. His neurological deficits had not improved. His family transferred him to hospice where he died within 24 hours.

## 2. Discussion

CPM, also called Osmotic Demyelination Syndrome (ODS), can present with altered mental status, dysarthria, or motor paresis. It was first described in 1959 and was attributed to alcoholism and hyponatremia [1]. Since then, guidelines have been established for sodium correction in hyponatremic patients which has improved mortality [2]. However, the most effective treatment for CPM is prevention. This is difficult because the pathophysiology of CPM is unclear and some patients are eunatremic with no rapid sodium correction [3].

One proposed mechanism of CPM is that neuronal demyelination begins when osmotically active substances such as sodium are depleted from the serum and free water shifts into the brain [4]. Over time, the brain adapts by distributing water into the cerebrospinal fluid and shifting intracellular solutes out of cells. If serum osmolarity then rises back to normal too rapidly, an osmotic gradient develops causing the destruction of myelin and the resulting death of neurons. The pons is less capable of transporting solutes across membranes than other areas of the brain leading to localized demyelination.

Only seven prior cases of CPM in AIDS patients have been reported. Those cases are atypical because most patients had either minimal hyponatremia or eunatremia and no evidence of rapid sodium correction [5]. In those cases, CPM was hypothesized to be secondary to lymphoma, Kaposi's sarcoma, hypoalbuminemia, opportunistic infections, or chemotherapy. Interestingly, another recent report describes a case of diffuse large B-cell lymphoma who presented with CPM and hypoalbuminemia but no other electrolyte abnormalities [6].

Our patient had minimal hyponatremia with hypoalbuminemia and lymphoma. It is plausible to hypothesize that some AIDS and cancer patients might be more sensitive to hyponatremia due to increased serum cytokines and metabolites associated with lymphoma or opportunistic infections. Furthermore, hypoalbuminemia was a common factor in both our case and the previously reported cases, possibly contributing to a hypoosmolar state in which CPM could develop. The mechanisms causing atypical CPM are unclear, and cases should be reported for further investigation.

In conclusion, it is important to consider CPM as a differential in any AIDS patient with altered mental status or neurological deficits. CPM in this population often has a unique presentation with minimal hyponatremia and no other electrolyte disturbance. Prevention remains the best treatment option given the high mortality rate and unclear pathophysiology. Tracking serum osmolarity in acutely ill AIDS patients may be a better marker for potential CPM than serum sodium and warrants further investigation.
